# Fast and behavior-selective exploitation of a marine fish targeted by anglers

**DOI:** 10.1038/srep38093

**Published:** 2016-12-06

**Authors:** Josep Alós, Miquel Palmer, Rosario Rosselló, Robert Arlinghaus

**Affiliations:** 1 Department of Biology and Ecology of Fishes, Leibniz-Institute of Freshwater Ecology and Inland Fisheries, Müggelseedamm 310, 12587 Berlin, Germany; 2 Instituto Mediterráneo de Estudios Avanzados, IMEDEA (CSIC-UIB). C/Miquel Marqués 21, 07190, Esporles, Illes Balears, Spain; 3 Division of Integrative Fisheries Management, Faculty of Life Sciences and Integrative Research Institute for the Transformation of Human-Environmental Systems (IRI THESys), Humboldt-Universität zu Berlin, Invalidenstrasse 42, 10155 Berlin, Germany

## Abstract

Harvesting of wild-living animals is often intensive and may selectively target heritable behavioral traits. We studied the exploitation dynamics and the vulnerability consequences of individual heterogeneity in movement-related behaviors in free-ranging pearly razorfish (*Xyrichthys novacula*). Using underwater-video recording, we firstly document a fast and high exploitation rate of about 60% of the adult population removed in just few days after the opening of the season. Subsequently, we tagged a sample of individuals with acoustic transmitters and studied whether behavioral traits were significant predictors of the vulnerability to angling. Tagged individuals revealed repeatable behaviors in several home range-related traits, suggesting the presence of spatial behavioral types. The individuals surviving the experimental fishery showed only localized and low-intensity movement patterns. Our study provides new insights for understanding the harvesting pressures and selective properties acting on behavioral traits of recreational fishing. Many fish stocks around the globe are today predominantly exploited by recreational fisheries. The fisheries-induced change in fish behavior described here may be therefore widespread, and has the potential to alter food-webs, profitability of the fisheries and to affect stock assessment by eroding catchability in the long-term.

Humans exploit wild-living animals by way of fishing and hunting since the origin of our species^1^. Because fish feature high in our society’s demand for food and recreation, many fish stocks show signs of overexploitation^2^, although many assessed stocks are beginning to recover or have already recovered in response to the implementation of proper management^3^. Most freshwater and some coastal fish stocks in developed countries are today predominantly exploited by recreational fisheries^4^. The role of recreational fishing in the global fishing crisis is now considered non-negligible^5^. However, the general lack of monitoring and assessment programs in recreational fisheries in many countries of the world imposes constraints on our ability to estimate exploitation rates^6^,^7^. This is potentially problematic because knowledge of current exploitation rate relative to the sustainable fishing mortality rate and the relation of current to unexploited spawning stock biomass are both crucial reference points, whose knowledge is essential for ensuring sustainable capture fisheries^8^.

In addition to the extraction of biomass, exploitation affects fish populations also by way of trait-selective harvesting^9^. There is now substantial evidence in the context of commercial fisheries that intensive and/or size-selective harvesting selects for fast life-histories^10^,^11^. Less evidence for fisheries-induced evolution (FIE) in recreational fisheries exist, but models^12^, observational studies^13^,^14^, and experiments^15^ have all demonstrated that fishing with hooks and lures can also generate similarly strong selection gradients than those observed in commercial fisheries. These findings are not surprising given that annual exploitation rates in recreational angling can be as high as 80%^16^. Although the economic consequences of FIE for fisheries may be not be severe when fisheries are managed sustainably^17^, the evolutionary consequences of recreational fishing will reduce the recovery ability of exploited stocks and the attractiveness of local fisheries to anglers and business^18^. Identifying the traits that render fish vulnerable to harvest are therefore of importance for ensuring sustainable exploitation^10^.

Vulnerability is a complex process in recreational fisheries. Behavioral traits have been suggested to play a major role, either due to selection directly acting on such traits or due to indirect selection responses emerging from correlation with life-history traits^19^,^20^. The argument underlying this assumption is that catching a fish with baited hooks, artificial lures, gill nets or traps strongly depends on the behavior-driven encounter probability and the active decision of a fish to attack or ingest the bait/lure or enter the trap or gill net^19^. Hence, individual heterogeneity in relation to behavioral traits such as boldness, space use, refuge seeking, energy acquisition (e.g., swimming activity) or aggression should play a major role in the vulnerability of fish to recreational fishing gear^21^, but few empirical studies on this topic exist so far^22^. Moreover, the scant evidence of behavior-selective fishing that exist has generally been generated from experimental settings in laboratories or in ponds (see ref. 23 for exception). Laboratory-based behavioral assays will rarely represent how the very same individuals behave in the wild^24^, and so far due to the difficulty of tracking fishes over long periods of time in the wild, only a few papers have documented consistent individual heterogeneity in fish behavior in free-ranging individuals targeted by fishers^23^,^25^,^26^.

With the development of fine-scale aquatic telemetry and the development of novel statistical tools applied to movement data such as state-space models (SSM), ecologists have now a powerful tool for studying individual behavioral heterogeneity *in situ*^27^ and how it correlates with the vulnerability of exploited wildlife and fishes^23^,^28^. One of the behavioral traits that we can measure in the field using positional data is the home range, i.e., the area used by an animal to fulfil its normal activities^29^. An individual’s home range is the outcome of a complex interplay of the environment and the intrinsic personality traits of the animals. Many animals develop home range and these can be highly predictable at the individual level but strongly vary among individuals^25^. The process behind a home range development is Brownian motion (i.e., movement according to random stimuli), but with a general tendency to remain around a specific area of interest (e.g., the center of the home range or a refuge, Fig. 1^30^). Any correlation between vulnerability and fish home range could have important ecological and evolutionary consequences by affecting the distribution of individuals and the spatial overlap of predators and prey^20^,^31^,^32^.

Harvested animals in systems where encounters with the human predator mainly determines the probability of capture are expected to display smaller home range areas and reduced exploration rates in response to exploitation in agreement with the recently proposed “timidity syndrome”^22^. Empirical evidence demonstrating these predictions are however both scant and inconsistent. While two studies^28^,^33^ demonstrated that hunters selects against elk, *Cervus elaphus* and pheasants, *Phasianus colchicus* that exhibit large home ranges, other recent work^23^,^34^ failed to find a relationship between fishing vulnerability and home range extension in Atlantic cod, *Gadus morhua* and the European lobster, *Homarus gammarus*, respectively. Simulation models have also shown the specific spatial patterns of harvesting by humans shapes the consistency and direction of selection gradients on home ranges^21^. By contrast, a consistent negative selection on how fast the individual explores the home range was uncovered in theoretical models where encounters strongly shape vulnerability^21^. There is a need to validate these predictions to understand the mechanisms behind the behavioral dimension of vulnerability.

The objectives of our work were two-fold. First, we sought to estimate the exploitation rate induced by recreational anglers on pearly razorfish *Xyrichtys novacula* (Linnaeus, 1758) using a before-after-impact-control (BACI) design based on underwater video cameras. Second, we used a novel SSM model applied to acoustic tracking data and investigated the individual heterogeneity in behavior and whether the home range behavior shown by individual fish was repeatable, hence representing a personality trait^25^. We then tested which individuals were harvested after the opening of the fishing season within a few days using a survival approach, and whether vulnerability to capture was related to the home range behavior.

## Results

### Recreational harvesting dynamics of pearly razorfish

The GLMM fitted to *n*Max as proxy of abundance of pearly razorfish demonstrated a significant (*p* < 0.05) interaction effect of area (no-take vs. exploited) × period (before vs. after the seasonal opening of the fishery, Fig. 2). The sign of the interaction effect indicated that the relative abundance of pearly razorfish strongly declined due to harvesting compared to the temporal trend in the unfished control area (Fig. 2). In general, the relative abundance of fishes present in the no-take control (ntMPA) area was much greater than in the exploited area (pMPA) as would be expected from an exploited equilibrium (Fig. 2). The abundance increased from the before to the after period in ntMPA due to natural fluctuations from a mean ± s.e. [CI] of 3.5 ± 0.16 [2.59, 4.85] individuals before to a value of 3.7 ± 0.16 [2.74, 5.06] individuals, but these differences in abundance were non-significant (Fig. 2). By contrast, the exploited area experienced a significant decrease in relative abundance from 1.05 ± 0.16 [0.77, 1.43] individuals to 0.47 ± 0.23 [0.29, 0.75] individuals after exploitation (Fig. 2). The dispersion of the residuals of the abundance model was estimated as 1.1. Thus, no evidence for over-dispersion was observed, suggesting a proper fit of the data using the Poisson distribution. Considering the slight temporal fluctuations in fish abundance observed in the control site and the significant decrease observed in the exploited population, we estimated a very rapid and intense exploitation rate of 57% [13.9, 79.2] over just three weeks in the pearly razorfish fishery.

### Individual heterogeneity and repeatability of home range behavior

The Bayesian SSM applied to the acoustic tracking detections (Fig. 3) revealed that tagged fish varied widely in their home range-related behaviors and distributed their home ranges across the entire array of omnidirectional receivers (Table 1). The means and s.d. for the home range-related behavioral traits were 217 ± 100 m for the variable *radius* and 0.006 ± 0.005 min^−1^ for the variable exploration rate (*k*). Overall, the acoustic tracking revealed strong among-individual differences in both mechanistic descriptors of home range suggesting high individual heterogeneity in home range behavior in our tagged sample of pearly razorfish. *Radius* and *k* were uncorrelated and therefore provided independent measures of the intensity (*k*) and spatial extent (*radius*) of the home range behavior (Supplementary Material Fig. SM1).

The estimated repeatabilities (*R*) to assess the consistency of individual heterogeneity in home range behavior over time in the five chosen daily-based behavioral traits were generally high (Fig. 4). We found latitude (*R*_*2012*_ = 0.97[0.93, 0.99] and *R*_*2013*_ = 0.93[0.82, 0.97]) and longitude (*R*_*2012*_ = 0.96[0.92, 0.99] and *R*_*2013*_ = 0.92[0.82, 0.97]) of the center of the activity to be highly repeatable in both study years, with *R* values close to 1. This indicated strong among-individual differences in the location of the center of the home range and in activity over the study periods in both years (Fig. 4 and Table 2). The daily space utilization approximated by the bivariate fixed-kernel utilization distributions (50% and 95% KUD in km^2^) was also repeatable, although values were slightly lower for the fish tagged in 2013 in relation to the 95% KUD: 50% KUD (*R*_*2012*_ = 0.78[0.61, 0.94] and *R*_2013_ = 0.81[0.57–0.91]) and 95% KUD (*R*_*2012*_ = 0.85[0.65, 0.95] and *R*_*2013*_ = 0.28[0.1, 0.56]). These results again indicated consistent differences in the spatial extent of the activity spaces across the two tracking periods (Fig. 4 and Table 2).

Finally, the *Rs* of the daily distance travelled also indicated strong among-individual differences in movement activity in both study years (*R*_*2012*_ = 0.93[0.8, 0.96] and *R*_*2013*_ = 0.74[0.55, 0.89]). These results overall suggested that the specific home range behavior shown by individual pearly razorfish can be interpreted as a repeatable behavioral trait, either being an outcome of personality or in the case of swimming activity a personality trait *per se*, thus demonstrating the existence of spatial behavioral types (Fig. 4). Conventional daily metrics of behavior of the pearly razor fish just described were highly positively correlated with the *radius* and *k* obtained by the mechanistic SSM-based home range approach (Supplementary Material Figs SM1 and SM2). Therefore, any result of the vulnerability analysis and posterior analysis of the selection gradients (see below) using the parameters of the mechanistic home range model described above can be extrapolated to these conventional behavioral metrics.

### Behavioral determinants of vulnerability in an exploited environment

**Figure 1:**
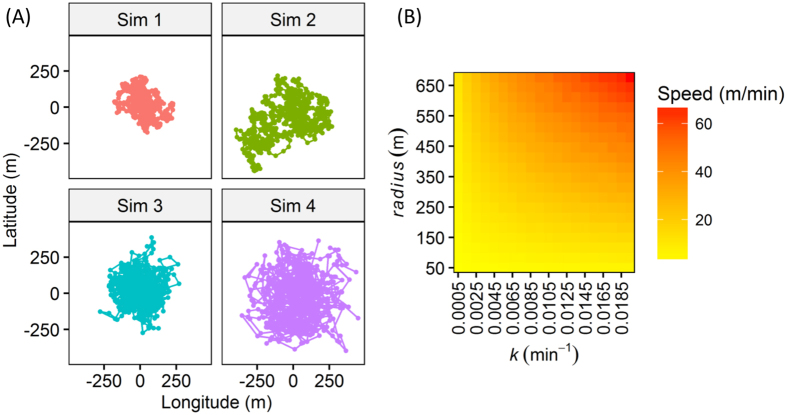
Home range behavior defined by an Ornstein–Uhlenbeck process. (**A**) Four discrete-time trajectories simulated to visualize the individual heterogeneity in the behavior of pearly razorfish, *Xyrichthys novacula* according to the home range model proposed by ref. 30. Four realistic combinations of the movement parameters (*k* and *radius*) in the range of empirically observed data (Simulation (Sim) 1: 0.001 min^−1^ and 245 m, Sim 2:0.001 min^−1^ and 387 m, Sim 3: 0.01 min^−1^ and 245 m and Sim 4: 0.01 min^−1^ and 387 m) were used to simulate 674 time-steps (of 15 min each) following the movement model. In all cases, the latitude and the longitude (in metres) of the center of the home range were 0. (**B**) Head map showing the effect of increasing the *radius* (in m) and the exploration rate (*k* in min^−1^) on the average swimming speed of the individual according to the movement model considered here. The increase in both parameters corresponds to an increase in the average swimming speed (m/s) of the individual.

In line with the results obtained in the BACI experiment using underwater video cameras, a large proportion of the tagged and acoustically tracked fish (68%) were harvested during a monitoring period of just 9 days after the opening of the harvesting season. After the AIC-reduction of the Cox model (see AIC-based ranking of all Cox models in Supplementary Material Table SM1), the only variables retained were the home range-related behavioral traits, *radius* and *k*, and both of them statistically affected the survivorship of fish (Table 3). Neither the spatial location of the home range, year, sex nor two-order interactions explained enough variance to be retained by the model (Supplementary Material Table SM1). The partial-effects survivorship plots show how, for example, the individuals characterized by a large value of the exploration intensity *k* were quickly removed from the fishery leaving behind fishes with smaller values for *k* (Fig. 5). Similarly, the individuals with large *radius* were quickly removed from the fishery (Fig. 5), overall suggesting that individual with larger swimming speed according to Fig. 1 were selectively harvested. No other variables were related to the survivorship of the tagged individuals, including gender/size, suggesting that vulnerability in a fished environment of our tagged sample of pearly razorfish was predominantly driven by home range and generally movement-related behavioral traits.

### Normalized selection strength on behavioral traits

The trait-specific mean-standardized selection gradients (*S*_*μ*_) were negative in all cases, collectively suggesting a reduced space use and smaller activity levels of the remaining individuals of our tagged sample after the harvesting peak. Specifically, *S*_*μ*_ was estimated as −1.43 for the parameter *radius* and −0.52 for the parameter exploration tendency *k*. Hence, selection by fishing was more strongly acting on the spatial extent compared to selection on exploration tendency. *S*_*μ*_ for the latitude and longitude of the center of the home range were also negative with values of −0.16 and −0.32, respectively. These findings collectively indicated recreational fishing-induced could favor selection towards individuals characterized by small home range and reduced exploration intensity with small swimming speeds.

## Discussion

Our work is one of the first assessments of the largely overlooked behavioral dimension of fish vulnerability to fishing gear directly measured in the wild^10^. Two of our findings are of particular importance. First, using fishery-independent methods we documented a fast and remarkably high recreational fishing mortality that removed nearly 60% of all available adult pearly razorfish population over a very confined temporal scale of just a few days. Second, our work is among the first to report a behavioral-mediated explanation for fish vulnerability acting on repeatable personality traits in the wild. Because the vulnerability to harvest was found independent of gender, and by the same token body size, our work supports theoretical^19^ and empirical laboratory evidence in crayfish, *Cherax destructor*^35^ that the mechanistic basis for vulnerability to passive fishing gear such as lures, hooks or traps is predominantly behavior-based rather than size-based. In fact, although positive size-selective vulnerability to angling has been commonly reported in the recreational fisheries literature^16^, we propose that the underlying mechanisms of vulnerability to fishing in many stocks will often be strongly or even exclusively behavior-based^19^.

There is the perception that recreational fishing is less efficient, and hence potentially has smaller extractive potential, compared to industrial marine fisheries^36^. However, due to cumulative effects in many coastal and freshwater stocks, recreational angling today is the major or single source of fisheries mortality in some coastal stocks^37^,^38^. In our study area we estimated the exploitation rate induced by anglers using fishery-independent data (underwater cameras) and a BACI design. Although exploitation rates could be directly estimated by mark and recapture studies observing tag returns of marked fishes by fishers^39^, estimating exploited biomass or abundance and associated reference points using fishery-dependent data notoriously difficult and costly^40^. In addition, conventional stock assessment approaches based on fishery-dependent data are prone to biases^41^,^42^. Our novel camera-based method is less sensitive to biases expected from fishery-dependent data (e.g., gear-selectivity, underreporting). Many coastal fish species are characterized by moving in confined home ranges and they form discrete meta-populations^43^. In these particular cases, underwater video cameras applied to exploited sites before and after a fishing peak could be easily implemented as a monitoring tool, especially in those coastal and inland areas intensively exploited by anglers with a lack of information on landings or tag return.

**Figure 2:**
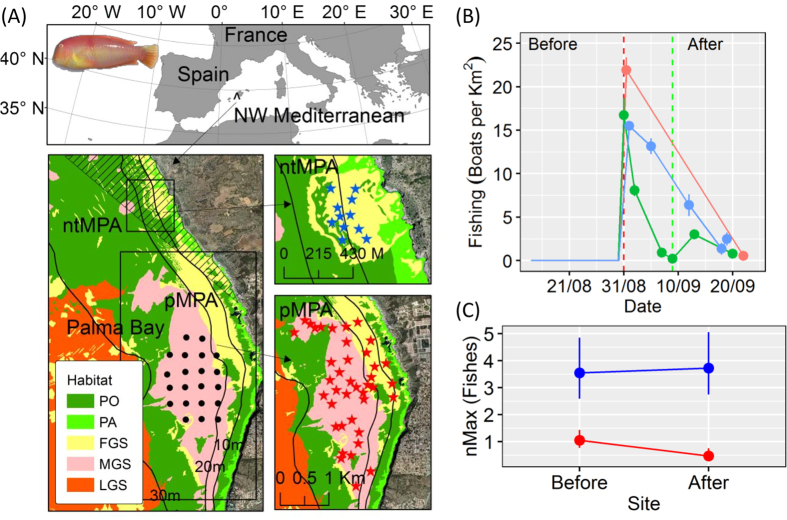
Location and dynamics of the recreational fishery of the pearly razorfish, *Xyrichthys novacula*. (**A**) Map of the study area located in the NW Mediterranean. The central map shows the study area located in the marine protected area (MPA) of Palma Bay, Balearic Islands, Mallorca, Spain (MPA delimited by the isobath of the 30 m in relation to land). The central map also shows the location of the no-take MPA (ntMPA in dashed), the partial MPA (pMPA) and the location of the 21 omnidirectional acoustic receivers (black dots) used for the tracking the fish. The right panels show a detailed map and the location of the sampling points (as stars) where the underwater video cameras were deployed within the ntMPA (in blue) and the pMPA (in red). The habitat of the study area was composed by seagrass of *Posidonia oceanica* (PO), photophilic algae habitats (PA), fine-grain sand (FGS), medium-grain sand (LGS) and large-grain sand (LGS). Note how the suitable habitat for the pearly razorfish (FGS and MGS) is surrounded by PO restricting the movement within this area and limiting dispersal. The map was created by the first author of the manuscript using ArcGis 10.3 for desktop (http://desktop.arcgis.com/es/desktop/) and self-created base maps and shapes (**B**) Average and standard deviation of the number of recreational boats per unit of area (boats × km^2^) in the pMPA (exploited area of ~6.5 km^2^) visually censed (every 15 min) during several days in 2012 (red), 2013 (green) and 2014 (blue). Before the opening the fishery is closed and enforced according to the seasonal closure stipulated for this species in the area. By September, 1 (vertical dashed red line) the fishery is opened, inducing a harvesting peak in the fishery. The survival of the pearly razorfish was assessed during this harvesting peak considering the first 9 days (vertical dashed blue line). (**C**) Main effects and confidence intervals (2.5% and 97.5%) of the generalized mixed effects model (GLMM) fitted to test the effect of the interaction between the study areas (no-take or control in blue vs exploited in red) and period (before vs after) on the abundance of the pearly razorfish (approximated by *n*Max, the maximum abundance of fishes observed on a video frame, see Methods).

Our method to assess the change in abundance due to fishing determined that in just a couple of days the abundance of pearly razorfish was reduced by close to 60%. In our fishery almost all annual fishing effort on the pearly razorfish in the study site is confined to a very limited temporal scale (Fig. 2). Hence, the “instantaneous” exploitation rates we estimated for the pearly razorfish can be considered annual mortality rates for this species. The annual exploitation rate reported here ranges among the highest ever reported for marine recreational fisheries^16^, and the total mortality of about 60% realized over just a few days mirrors the entire annual fishing mortality estimated for Atlantic cod exploited by anglers in southern Norway^44^. Although we have no estimate of *F*_*MSY*_ at our disposal, the sharp abundance decline in the exploited site after only few days of fishing relative to the control site suggests the population could be close to experiencing recruitment overfishing, which often occurs when the spawning stock is reduced to below 35% of the virgin stock^45^. The exploitation rate is, however, only one component assessing the sustainability of the fish stock, and one has to also provide data on the way recruitment happens in the focal fishery from spawners thriving elsewhere along the coast^8^. It is our hope that our work stimulates the development of assessments and related population dynamics models that take into account the fast and high exploitation rate caused by anglers obtained here. It is only then that we can finally conclude whether the fast and high exploitation rate documented here is sustainable or not.

We found that angler-induced exploitation of pearly razorfish is not only fast and intensive, but may also be non-random with respect to behavioral traits as revealed by our vulnerability experiment. The major limitation of our work is that we do have an unbiased sample of movement behaviors of the population of razorfish. Instead, our vulnerability analysis was based on a subset of fish behavioral types, given that all of the tagged fishes were captured by angling in the first place. No gear is entirely unselective for behavioral traits, which is why it is impossible to create a random sample of behavioral types. Nevertheless, it is very likely that the behavioral types we studied represented a subset of the random population. Accordingly, the selection gradients we estimated on behavioral traits are unlikely to represent the selection pressures at the population level. Despite this limitation, the selection portion of our study provides strong evidence for the mechanistic basis of angling vulnerability, suggesting that corresponding selection pressures are likely to exist in nature. Moreover, we measured individual heterogeneity of behavioral traits and vulnerability to harvest in the wild, adding realism to the study findings. All of the existing studies on the relationships of behavioral traits and vulnerability to capture have either been entirely conducted in the laboratory^35^ or in semi-natural ponds^46^ or have tested wild-captured fish for personality in laboratory settings^47^,^48^,^49^. However, there is an increasing evidence that laboratory-assayed behavior is not consistent with what the very same animals do in the wild and hence there is a need for testing relationships among behavior and fitness in a natural contexts^24^. This fact highlights the relevance of our findings as they are closer to the reality of the system.

We specifically have found that movement parameters characterizing the extension of the home range and the exploration rate were the only predictors of the survivorship of the tracked individuals after the opening of the fishery. Collectively, individuals with small foraging areas and exploration rates and with low average swimming speeds survived the harvesting season. This pattern was consistent across the two years and independent of size/gender. Moreover, we found high repeatability of the daily home range-related behaviors. There is growing evidence about consistent (i.e., context-and time-independent) individual heterogeneity in behavioral traits leading to personality traits that form behavioral types^50^. Our study, however, has revealed values of *R*s for some daily movement metrics (daily space use, center of activity and travelled distance) that were higher than those reported in previous telemetry studies in the wild^23^,^25^. The limited body size of the pearly razorfish required the use of very small acoustic tags (pico-tags), with a life-span of only a few days (Table 1). Moreover, acoustic tracking and in general any telemetry studies are constrained to the limited sample size due to the high cost of the technology. Therefore, it is possible that our *Rs* values were overestimated due to the limitations in the duration of tracking and the potential temporal autocorrelation of the behaviors measured at daily scales^51^ and we thus have to be cautions with over-interpreting the exceedingly high *R*s. However, a meta-analysis on animal personality reported average *R* of 0.37 among 98 species across several taxa^52^, which were estimated by on average 4.4 repeated observations of behaviors. The data in our work exceed in most of the cases these average values. We therefore feel that it is a safe conclusion that home range behavior is an expression of the underlying personality of the pearly razorfish in agreement with other recent findings in other species^23^,^25^, conforming spatial behavioral types that are linked to the vulnerability of the individual to be harvested.

We concluded from our previous simulation work^21^ that consistent selection for low activity phenotypes should be expected across fishing styles, while the selection gradient on home range size could range from positive to negative depending on the fishery and the inherent stochastic properties of fish movement. In this previous theoretical paper we simulated among-individual variability according to a mean and s.d. of 200 ± 20 m of the size of the home range. Our new empirical data in the pearly razorfish suggests that this variability is actually much higher (217 ± 100 m of the size of the home range) defining a much broader gradient of spatial behavioral types. The large behavioral gradient, and the fact that larger space uses increase the spatial overlap between the fish and fishers and thus the encounter probability among them just by chance is likely the cause of why we found a negative selection gradient acting on spatial extension of the home range in our fishery system. We also found that individuals characterized by a large exploration metric (*k*) were more quickly harvested from the fishery, generating an estimate of a negative selection gradient on *k*, in agreement with our previous theoretical work^21^. The selection gradients that we estimated for *radius* and *k* was found to be high compared with published values for a range of traits under natural selection, despite our sample being biased towards angled fish^53^. Our results therefore suggest the potential for recreational fishing favoring individuals with low swimming speeds and contrived space, which could conform part of the timidity syndrome at population level recently described for exploited systems^22^.

A recent meta-analysis suggests that about 52% behavioral variation across taxa is attributable to additive genetic variation^54^. Our work therefore demonstrates that the potential for FIE of behavioral traits certainly exists. Further work should therefore move forward exploring whether the population is actually responding this selective properties of recreational fishing and individuals of the population are decreasing their space use while accounting for plastic responses. Until population-level data are available, we can only speculate about a number of potential consequences of our findings. Recent work in largemouth bass, *Micropterus salmoides* protected from fishing also shows that exploited bass are more timid, which could have been caused by both evolution and learning similar to the case in pearly razorfish^55^. If pearly razorfish become increasingly difficult to capture, this behaviorally-related mechanism will lower population-level catchability, which affects stock assessments and angling quality^42^. Moreover, behavioral traits such as exploration are usually positively correlated with as growth rates and thus increased timidity and contrived space use could contribute to the downsizing of adults^13^. Fortunately, the quickly diminishing fishing effort on the pearly razorfish after just three weeks of angling is evidence of a somewhat self-regulating fishery where anglers leave as abundance of vulnerable fish and catch rates drop. This behavioral-mediated dynamic interplay of the pearly razorfish and anglers should be studied further in detail, in particular as relates to the replenishment dynamics of the adult stock across years, which could contribute to the fishery being sustainable even at the high exploitation rates reported here. Considering the growing intensity by which many freshwater and marine systems are being exploited by recreational gears^38^, we propose that the selective exploitation on movement behavioral traits may be widespread and can impact many different aspects of ecosystem functioning and ecosystems services provided by wildlife and fishes.

## Methods

Methods were carried out in accordance with approved guidelines. Specifically, all experimental protocols, including fish capturing and tagging, were approved by the authority responsible for marine natural resources of the study area (Fisheries Department of the Balearic Island), through a permit to the CONFLICT Project (ref: CGL2008-00958) and to the REC2 Project (ref: CTM2011-23835).

### Study case: the recreational fishery of the pearly razorfish

We studied the pearly razorfish population harvested in a local fishery located in the Balearic Islands (Fig. 2 and see ref. 56). The pearly razorfish is the main target of the fishery, it is very popular in the Mediterranean, and the fishery is based on hook-and-line gear baited with natural baits (usually live shrimp). The fishery is located within a marine protected area (MPA) where two main areas with different levels of protection are stipulated (Fig. 1): a no-take MPA (ntMPA) where all fishing activities are prohibited and a partial MPA (pMPA) where fishing for the pearly razorfish is permitted outside a closed season (Fig. 2). All pearly razorfish fisheries of the Balearic Islands remain closed from April, 1 to August, 31 according to local legislation. This fact not only creates a temporally clustered socially-related harvesting boost (Fig. 2), but also allows observing fish density and fish behavior before, across the exploitation phase and after the fishery opening in both the fished and protected control area. The peculiar habitat configuration of the fishery strongly limits the dispersal of adult fishes (who do not cross extended seagrass beds, Fig. 2), and the management of the fishery offered us a unique opportunity for testing our hypotheses.

### Estimation of exploitation rate

We randomly defined 49 sampling points within the MPA of Palma Bay (n = 11 in ntMPA and n = 38 in the pMPA) (Fig. 2). We used a fishery-independent method for assessing the abundance of the pearly razorfish just before and after the harvesting peak using a custom device to which a baited underwater video camera (Model GoPro Inc., © 2014) was attached. The video camera was deployed for a minimum of 30 min in each of the sampling points, and we used *n*Max as proxy of real abundance, which is defined as the maximum number of individuals observed in a single frame during the viewing interval^57^. We fitted a Poisson-GLMM in the R-package^58^, where *n*MAX was regressed on the interaction effect (the effect if interest) of site (no-take vs. exploited) × period (before vs. after) with sampling site ID considered as random effect (over-dispersion was checked). We discarded spatial pseudo-replication of the sampling points by fitting the univariate Moran’s I autocorrelation. The estimated interaction effect was then used to calculate the exploitation rate while controlling for the environmental-related changes in the abundance by comparing exploited and control (baseline) sites.

### Individual heterogeneity in the movement behavior of the pearly razorfish

We used the mechanistic approach proposed by ref. 30 to assess the individual heterogeneity of home range-related behaviors in the wild based on the integration of a movement model (a random walk defined by an Ornstein–Uhlenbeck process) into a Bayesian SSM using acoustic individual tracking data. According to the SSM proposed by ref. 30, four mechanistic behavioral parameters that lead the establishment of home range areas were estimated at the individual level: the size of the circular home range (*radius*) in meters as a surrogate of the total foraging area and activity space, the harmonic force (exploration rate *k* in min^−1^) that represents how strongly a fish is attracted toward the center of its home range, i.e., how fast and intense the home range is explored, and the latitude and longitude of the center of the home range. Figure 1 shows four fish trajectories simulated from our movement model to visualize how individual heterogeneity in *radius* and *k* affects the fish trajectory and the average daily swimming speed.

**Figure 3:**
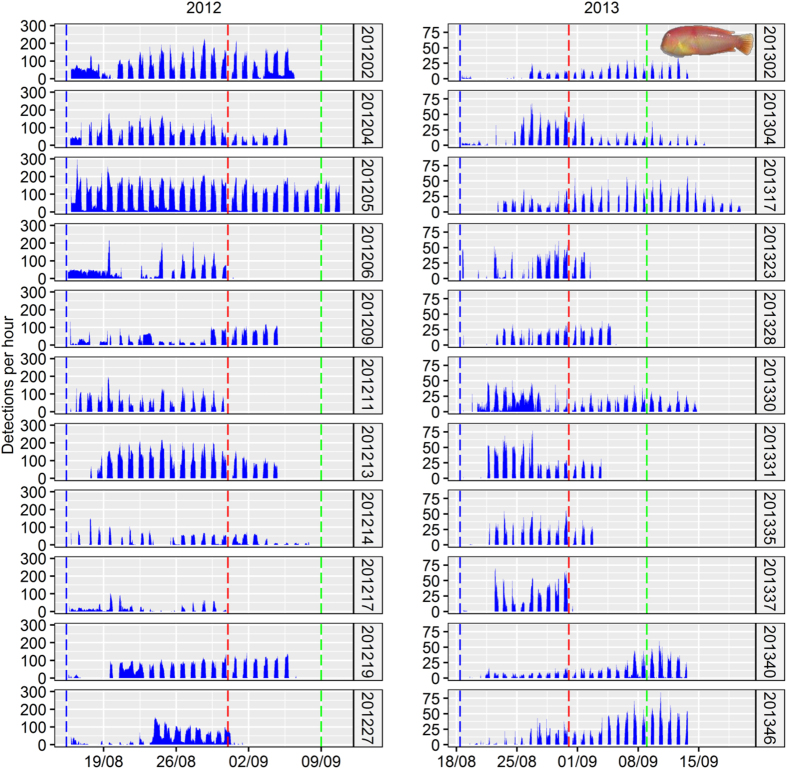
Miniaturized acoustic tracking of the pearly razorfish, *Xyrichthys novacula* in the wild using an array of omnidirectional receivers Chronograms of the number of acoustic detections per hour of the fish tagged and tracked in our study area in the years 2012 and 2013. The plot shows the day of the tagging (vertical blue line), the opening of the fishery (red line) and the end of the monitoring period for assessing survival to the fishery (green line). Note the pattern of detections generated by the pearly razorfish characterized by the lack of detections during the night as the individuals bury in the soft bottom to avoid predation and to rest.

**Figure 4:**
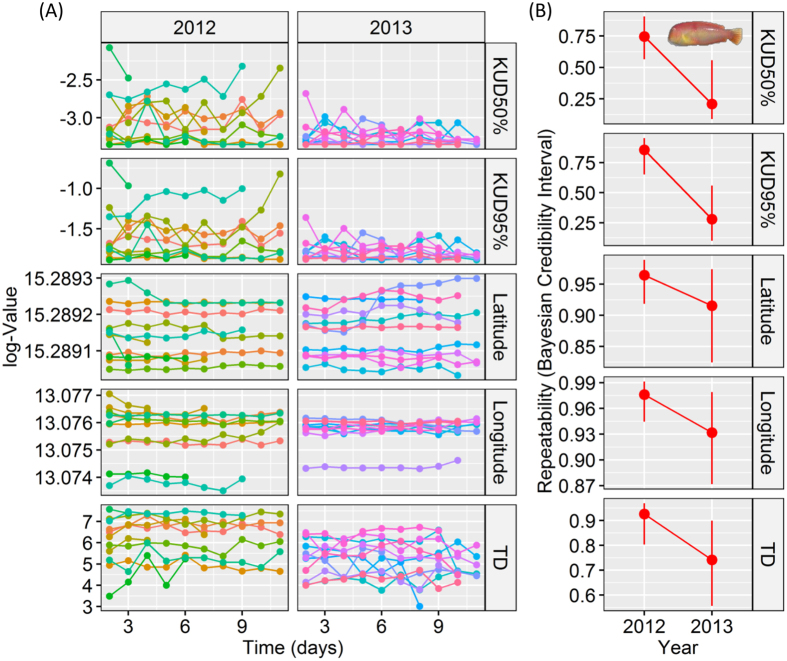
Individual heterogeneity of home range-related behaviors and the existence of behavioral types in the pearly razorfish, *Xyrichthys novacula*. (**A**) Daily values (natural logarithm transformed) of the space use, center of activity and total distance travelled (TD) in the pearly razorfish revealed by aquatic telemetry. Each color represents an individual, and the Julian days were transformed and standardized to ordinary numbers for improved visualization. The sample size (days) was on average across all individuals 10.8 ± 3.5 days. The left panel shows the daily space use as 50% and 95% KUD in km^2^, the latitude and longitude of the center of daily activity in geographic coordinates and the total distance travelled (in m) on a daily basis for each of the pearly razorfish tracked in 2012 and 2013. Note the small within- and the consistent among-indivuidual variability in all behavioural traits. (**B**) Repeatability for each trait and year and its Bayesian Credibility Intervals (BCI, 2.5% and 97.5%).

**Figure 5:**
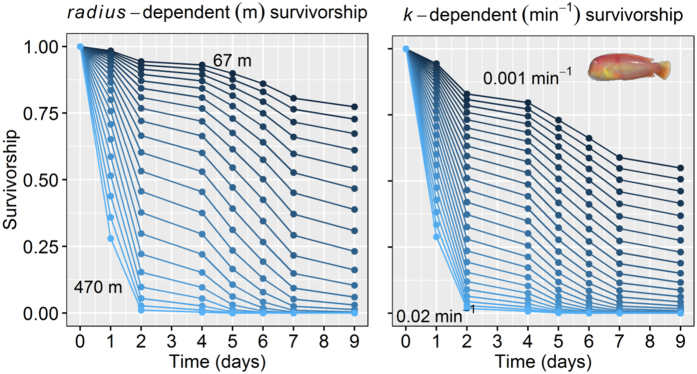
The fitness-consequences of harvesting on behavioral types in the pearly razorfish, *Xyrichthys novacula*. Predicted survivorship from the Cox-regression model fitted to test the effect of *radius* and exploration rate *k* on the survival (as surrogated of fitness in the fished environment). The survivorship plots show the predicted survivorship of 21 simulated individuals from the minimum and maximum *radius* (in m) observed in our data with an increment of 20 m. In case of *k*, the plot shows the predictions for 25 individuals from the minimum and maximum of *k* (in min^−1^) values observed in our data by incremental steps of 0.001 min^−1^. The average effects in both cases were statistically significant as revealed by the Cox-regression.

To feed the SSM with field data, we carried out a standard acoustic tracking experiment in the study area (Fig. 2). In August 2012 and 2013 before the opening of the harvesting season wild pearly razorfish were captured by angling, tagged using tiny acoustic tags of the model Pico-Tag-2 (Sonotronics©) and tracked using an array of omni-directional receivers (Table 1, Figs 2 and 3). The details of the tagging procedures are explained in ref. 56. The only practically possible, non-invasive method to capture live pearly razorfish is hook-and-line. Therefore, the estimation of the individual heterogeneity in the movement behavior, as well as the study of the relationship between behavior and vulnerability (see below) was based on angled fish. The main aim of our study was to provide evidence for spatial behavioral types and its relationship with vulnerability. Although the variability of behavioral types was likely biased towards fish that are all in general vulnerable to angling gear, our approach was reasonable to study the mechanistic behavioral basis of vulnerability in the wild. However, results on this portion of the study cannot be up-scaled to the whole population. After cleaning the data-base following the decision criteria tree for differentiating among false and spurious detections proposed by ref. 56, the data was composed of 22 daily time-series of acoustic detections of individual fish (n = 11 fish in 2012 and n = 11 fish in 2013) tagged before the opening of the fishery and tracked across the exploitation peak after the opening of the fishery (Fig. 3). The temporal series of acoustic detections were fitted to a SSM according to ref. 30 to estimate the four home range movement parameters (*radius*, *k*, latitude and longitude) at the individual level. Three Markov chain Monte Carlo (MCMC) were run assuming minimal prior knowledge^30^, and the convergence of the MCMC chains of all parameters was assessed by visual inspection of the plots of the iterations and tested using the Gelman-Rubin Statistic^59^. The posterior distribution of each movement parameter was used to characterize each pearly razorfish behaviorally (i.e., parameters were used as traits).

### Repeatability of movement behavior

The repeatability score (*R*) assesses the degree of consistency of behaviors shown by individuals over time^60^. *R* represents the phenotypic variation attributable to individual heterogeneity and is often used to characterized animal personalities; it can be estimated as:


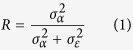


where 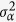 is the between-individual variance and 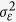 is the within-individual variance for a given behavioral trait.

Our mechanistic approach to the home range behavior summarized the behavior of fish in relation to four movement parameters, which was useful for testing our vulnerability hypothesis. However, this approach did not provide repeated measures for studying how consistent and repeatable the four behaviors were over time. To obtain alternative repeated measures and estimate *R*, we calculated three conventional home range-based metrics on a daily basis using positional data similar to ref. 25. We first calculated the daily space use defined as the area (in km^2^) using the 95% and 50% KUD in the same way as ref. 56. Secondly, we calculated the latitude and the longitude of the daily activity for each fish as the average of the latitude and the longitude used data for each day. Thirdly, we estimated the total distance travelled (in m per day) using the summation of the Euclidean distances between all two consecutive positions. MCMC-GLMM was used to decompose the raw phenotypic variance of each single behavioral metric into between- and within-individual variances, and *R* was computed following equation 1 using the library *MCMCglmm* of the R-package^51^. The distribution of the residuals was inspected for proper fit of the data, and a transformation (natural logarithm) was applied in the cases were the normality in the residuals was strongly deviating from a normal distribution.

### Behavioral determinants of vulnerability in a fished environment

To examine whether home range behavior and other traits (e.g., body size or gender) were correlated with vulnerability (i.e., mortality in an exploited environment), a survival model was fitted to the survivorship data of the tagged fish. Survival of each tagged fish was evaluated across/during the intensive harvesting period of 9 days, which covered the first two weekends after the opening of the fishery that concentrated most of the fishing pressure of the year according to the effort dynamics of the fishery (see Figs 2 and 3 and Table 1). Moreover, this monitoring period was chosen to keep the overall monitoring period from before to after fishing to within 23 days to avoid reaching the life-span of the small acoustic tags that we used^56^. We assumed that the individual was harvested when the particular animal did no longer follow the normal diel behavior defined in ref. 56. No tag loss was observed in the 22 fish according to the characteristic flat pattern of acoustic detections generated by tag loss described in our study area^56^. Any disappearing signal rendered harvest the most likely outcome for the individual as the last position (likely location of fish when was captured) was clearly within the array of omni-directional receivers for all individuals that we tracked (see Supplementary Material Fig. SM3). Note that the potential for tagging mortality was diagnosed following the decision criteria for acoustic tracking developed by ref. 56, and natural mortality is highly unlikely during for this short time scale. For all of these reasons, it was reasonable to assume that the fish that were lost during the sampled period of 9 days were harvested by anglers.

We fitted a Cox- regression model to the binary (harvested or not) and continuous (survival days) response data using the *survival* library of the R-package. The full model included the individual mechanistic movement-related behavioral traits (*radius*, *k* and the latitude and longitude of the center of the home range), the study year, gender and all of the second order interaction with gender. Fish length was strongly correlated with gender as the pearly razorfish is a protogynous hermaphrodite, and we selected gender to be incorporated in the Cox model. The coefficients of the Cox regression were estimated for the minimally adequate model ranked by the Akaike information criterion (AIC). The predicted survivorship probabilities over the exploitation phase were plotted using the function *survfit* of the R-package for significant effects.

### Selection gradients on behavioral traits

We calculated the mean-standardized selection gradient (*S*_*μ*_) acting on each movement trait according to ref. 61 as follows:


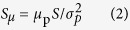


where *S* is the selection gradient defined as the difference between the phenotypic mean of all tagged individuals and the mean of survivals, and *μ*_P_ and 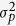
are the mean and the variance of the movement trait of all tagged individuals prior to the harvesting impact^21^. Note that *S*_*μ*_ is a clean measure of selection strength and allows ranking the strength of selection acting on each of the various behavioral traits independent of the trait’s mean and variance^61^. *S*_*μ*_ can be interpreted as an elasticity of fitness to trait change^61^. For example, an *S*_*μ*_ = 0.5 means that doubling the trait value elevates fitness by 50%.

## Additional Information

**How to cite this article**: Alós, J. *et al*. Fast and behavior-selective exploitation of a marine fish targeted by anglers. *Sci. Rep.* **6**, 38093; doi: 10.1038/srep38093 (2016).

**Publisher's note:** Springer Nature remains neutral with regard to jurisdictional claims in published maps and institutional affiliations.

## Supplementary Material

Supplementary Information

## Figures and Tables

**Table 1:** Summary of the characteristics of the pearly razorfish, *Xyrichthys novacula* electronically tracked to test behavioral-related fitness in our survival approach.

ID	Fish size (mm)	Gender	Year	Detections	Positions	Survival days	Captured	Latitude (m)		Longitude (m)		*k* (min^−1^)		*Radius* (m)	
								Mean	s.d	Mean	s.d	Mean	s.d	Mean	s.d
201202	160	Female	2012	14222	506	7	1	4365343	13	477000	13	0.008	0.002	168	12
201204	190	Male	2012	13924	609	6	1	4364851	18	477476	17	0.007	0.001	233	16
201205	220	Male	2012	26678	700	9	0	4365453	7	477343	8	0.003	0.001	67	8
201206	179	Male	2012	5261	243	2	1	4364763	41	477504	40	0.006	0.001	289	31
201209	189	Male	2012	2142	150	5	1	4365043	90	477682	80	0.002	0.001	260	51
201211	185	Male	2012	8699	477	1	1	4365119	33	477091	33	0.006	0.001	355	27
201213	182	Male	2012	19281	584	5	1	4364668	14	477389	14	0.004	0.001	138	14
201214	184	Male	2012	994	73	9	1	4364789	39	476441	35	0.010	0.007	138	32
201217	163	Female	2012	1002	60	1	1	4364893	98	477380	98	0.010	0.003	470	48
201219	162	Female	2012	9484	466	7	1	4365472	69	477459	69	0.001	0.000	211	47
201227	160	Female	2012	3954	383	2	1	4365064	34	476297	33	0.008	0.001	380	27
201302	159	Female	2013	1662	375	9	0	4365280	39	477293	44	0.003	0.002	164	49
201304	181	Female	2013	2833	323	9	0	4364662	17	477230	17	0.010	0.003	180	20
201317	167	Male	2013	2739	345	9	0	4364914	21	477229	22	0.006	0.003	182	22
201323	183	Male	2013	2483	289	2	1	4365499	10	477315	10	0.011	0.006	82	19
201328	176	Male	2013	2557	421	6	1	4365749	112	477271	94	0.001	0.000	296	39
201330	174	Male	2013	1556	285	9	0	4365226	152	476663	161	0.001	0.001	236	72
201331	157	Female	2013	3973	471	4	1	4364782	61	477274	61	0.001	0.001	265	42
201335	153	Female	2013	2488	338	2	1	4364761	21	477287	22	0.008	0.002	209	22
201337	147	Female	2013	2910	338	1	1	4364800	11	477229	12	0.025	0.009	169	23
201340	193	Male	2013	2507	351	9	0	4365498	37	477338	38	0.003	0.001	200	33
201346	194	Male	2013	3664	350	9	0	4365155	19	477363	17	0.004	0.002	79	22

The table shows characteristics of the tagged fish including fish ID, gender, fish size (total length in mm), the tracking year, the number of acoustic detections used for the State-Space Model (SSM), the total number of positions estimated and the fate of the individual during the monitoring survival period (captured = 1 or not = 0, and the survival days since the opening of the fishery). The table also shows the posterior distribution (Bayesian mean and s.d.) of the movement parameters (latitude and longitude coordinates in Universal Transverse Mercator (UTM, zone 31S) of the center of the home range, *radius* and exploration rate *k*) estimated using the SSM approach.

**Table 2:** Mean and s.d. of the personality-related daily behavioral metrics estimated in the pearly razorfish, *Xyrichthys novacula* using aquatic telemetry: Daily space use (50% and 95% KUD in km^2^), the coordinates (latitude and longitude) of the center of daily activity and the total daily distance travelled (in m).

ID	Space use (km^2^)				Center of activity (Coordinates)				Travelled daily distance (m)	
	KUD95%		KUD50%		Latitude		Longitude		Distance	
	Mean	s.d	Mean	s.d	Mean	s.d	Mean	s.d	Mean	s.d
201202	0.20	0.02	0.05	0.01	4365344	24.49	477002	36.33	809.7	162.9
201204	0.22	0.03	0.05	0.01	4364849	30.81	477473	67.02	1036.1	292.3
201205	0.16	0.00	0.04	0.00	4365453	12.36	477340	22.42	130.7	36.2
201206	0.22	0.03	0.05	0.01	4364752	44.29	477486	142.04	913.5	260.8
201209	0.17	0.01	0.04	0.00	4365026	38.96	477753	151.13	374.7	116.1
201211	0.26	0.08	0.06	0.02	4365118	67.65	477086	128.14	1373.9	341.2
201213	0.17	0.01	0.04	0.00	4364676	23.28	477398	61.92	362.6	94.4
201214	0.16	0.01	0.04	0.00	4364816	53.14	476436	28.59	132.5	91.5
201217	0.39	0.11	0.10	0.03	4364942	216.77	477468	154.31	1496.0	532.0
201219	0.17	0.02	0.04	0.01	4365517	102.48	477479	17.97	178.6	87.6
201227	0.32	0.04	0.07	0.01	4365067	35.60	476296	80.04	1520.1	237.7
201302	0.16	0.01	0.04	0.00	4365272	57.36	477286	14.99	89.7	44.4
201304	0.18	0.02	0.04	0.00	4364658	40.17	477236	51.22	431.4	176.7
201317	0.17	0.02	0.04	0.01	4364911	35.62	477231	56.49	255.1	88.2
201323	0.16	0.01	0.04	0.00	4365517	50.95	477304	39.17	165.3	71.3
201328	0.17	0.02	0.04	0.00	4365493	289.39	477324	92.05	143.5	81.6
201330	0.16	0.00	0.04	0.00	4365337	64.07	476572	43.27	87.6	27.0
201331	0.17	0.01	0.04	0.00	4364804	66.22	477282	105.25	223.1	80.8
201335	0.19	0.03	0.04	0.01	4364762	50.05	477282	46.17	446.8	147.2
201337	0.18	0.01	0.04	0.00	4364802	20.70	477231	22.39	703.4	156.9
201340	0.17	0.01	0.04	0.00	4365494	79.14	477338	35.65	244.0	103.4
201346	0.16	0.00	0.04	0.00	4365160	18.29	477367	19.18	74.7	18.7

ID refers the identification number of the fish.

**Table 3:** Results of the survival analysis (Cox regression model) testing the effects of different variables on the probability of a pearly razorfish, *Xyrichthys novacula*, to be harvested.

*Minimal Adequate Model*	coef	exp(coef)	s.e.(coef)	z-value	Pr(>|z|)	
Exploration *k*	0.019	1.02[1.001, 1.03]	0.006	3.82	<0.001	***
*Radius*	0.009	1.01[1.008, 1.02]	0.003	5.09	<0.001	***
AIC = 66.7 (AIC null model = 79.9)						
*r*^2^ = 0.52						

The table shows the regression coefficients (coef) of the logarithm of the hazard ratio and its standard error (s.e.), the exponent of the coefficient for interpretation, the z-value and p-value of the full model and the minimal adequate survival model after AIC-based optimization. The full model included the behavioral metrics (*radius* and exploration *k* and the location of the center of the home range), gender, year and all second-order interaction with gender. Only the movement behavioral metrics (*radius* and exploration *k*) were retained by the optimal survival model and in both cases were significant predictors of harvest.

^***^Very high significant effect.
