# Supplementary material for: Fast and behavior-selective exploitation of a marine fish targeted by anglers
Source: Sci Rep. 2016 Dec 6;6:38093. doi: 10.1038/srep38093 (PMC5138602; doi:10.1038/srep38093)
Supplement: Supplementary Information [file srep38093-s1.pdf]

Supplementary Material for the manuscript entitled:

Fast and behavior-selective exploitation of a marine fish targeted by anglers

By Josep Alós, Miquel Palmer, Rosario Rosselló and Robert Arlinghaus

Contents:

Figure SM1: Two-paired correlations among the different space use variables estimated using the state-space model and daily behavioral metrics.

Figure SM2: Two-paired correlations among the locations of the home range estimated using the state-space model and daily behavioral metrics.

Figure SM3: Plot of the last position of the captured individuals of tagged pearly razorfish within the array of omni-directional receivers.

Table SM1 Ranking of Cox regression model testing the effects of different variables on the probability (Survival days and capture) of a pearly razorfish to be harvested models based on AIC-based optimization.

Figure SM1 Two-paired correlations among the behavioral-movement parameters estimated using the State-space model (Radius in m and exploration-*k* in min^{-1}) and the daily behavioral metrics estimated in the pearly razorfish using aquatic telemetry [daily space use (50% and 95% kernel utilization distributions, KUD in km^2] and the total daily distance travelled in m). The points of the plot correspond to the individual mean value for each parameter. The Pearson correlation coefficient (or covariance) is shown as numbers and the level of significance with stars.

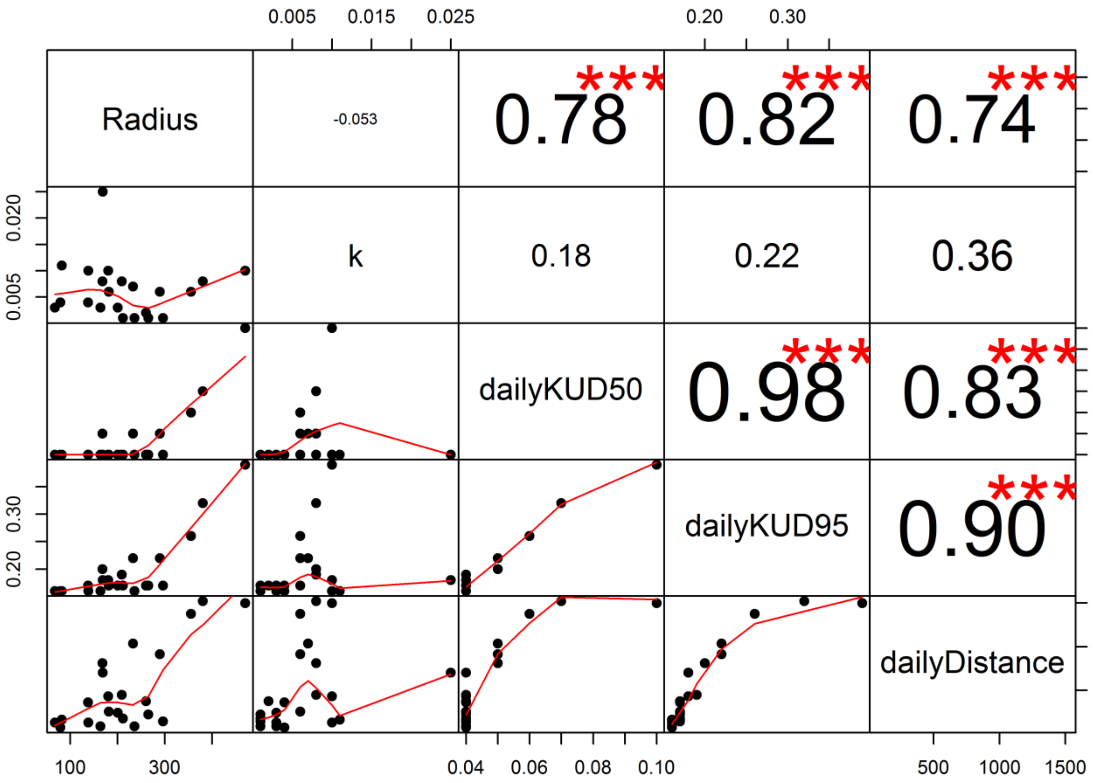

Figure SM2 Two-paired correlations among the location of the center of the home range estimated using the State-space model (latitude and longitude in geographic coordinates in UTM) and the personality-related daily center of activity (latitude and longitude in geographic coordinates in UTM). Points of the plot correspond to the mean value for each metric. The Pearson correlation coefficient (or covariance) is shown as numbers and the level of significance with stars.

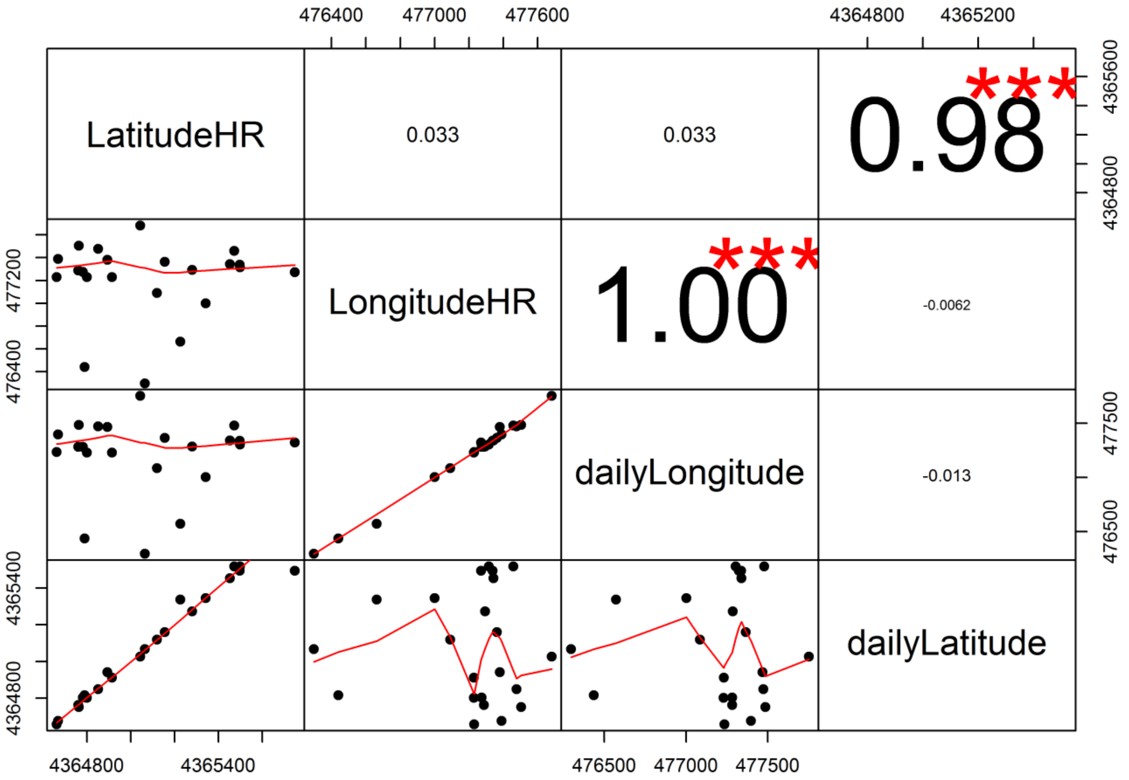

Figure SM3 (A) Location of the receivers array in the south of Mallorca Island, NW Mediterranean. (B) Array of omni-directional receivers (black circles) as well as the detection range (300 m as an empty circle, the maximum detection range is ca. 1000m). The last position when the individuals were theoretically captured is plotted as colored circles and labeled with the identification id. (C) One of the omnidirectional receivers moored in our experimental area. The map was created by the first author of the manuscript using ArcGis 10.3 for desktop (http://desktop.arcgis.com/es/desktop/) and self-created base maps and shapes.

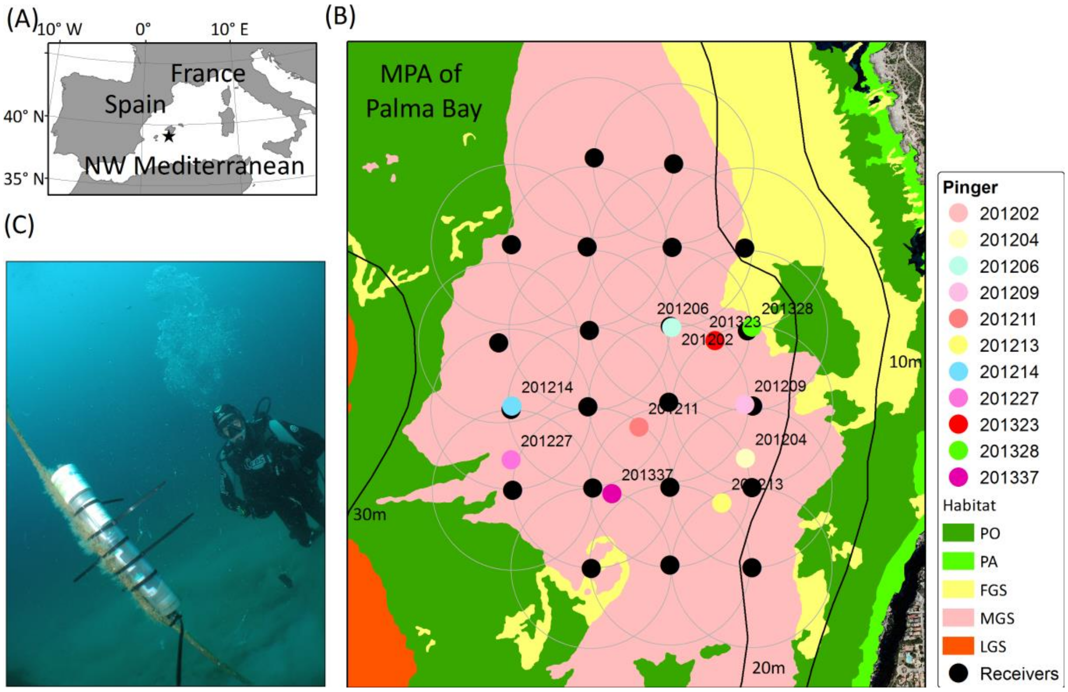

Table SM1 Ranking of Cox regression model testing the effects of different variables on the probability (Survival days and capture) of a pearly razorfish to be harvested models based on AIC-based optimization. The AIC of the model as well as the increment (ΔAIC) for each model are shown. The Minimal adequate model is highlighted in bold.

Model: (Survival days, capture) ~	AIC	ΔAIC
**1) Exploration *k* + *Radius* (Minimal adequate model)**	66.7	0
2) Exploration *k* + *Radius* + Longitude	66.99	0.29
3) Exploration *k* + *Radius* + Longitude + Sex	68.77	2.07
4) Exploration *k* + *Radius* + Longitude + Sex + (Sex × Exploration *k*)	69.67	2.97
5) Exploration *k* + *Radius* + Longitude + Latitude + Sex + (Sex × Exploration *k*)	70.61	3.92
6) Exploration *k* + *Radius* + Longitude + Latitude + Sex + (Sex × Exploration *k*) + (Sex × *Radius*)	71.94	5.24
7) Exploration *k* + *Radius* + Longitude + Latitude + Sex + Year + (Sex × Exploration *k*) + (Sex × *Radius*)	73.48	6.78
8) Exploration *k* + *Radius* + Longitude + Latitude + Sex + Year + (Sex × Exploration *k*) + (Sex × *Radius*) + (Sex × *Latitude*)	74.96	8.26
9) Exploration *k* + *Radius* + Longitude + Latitude + Sex + Year + (Sex × Exploration *k*) + (Sex × *Radius*) + (Sex × *Latitude*)	77	10.3
